# Primary Renal Angiosarcoma Mimicking Renal Cell Carcinoma: A Case Report

**DOI:** 10.7759/cureus.3841

**Published:** 2019-01-07

**Authors:** Danny Darlington, Fatima Shirly Anitha

**Affiliations:** 1 Urology, Government Stanley Medical College and Hospital, Chennai, IND; 2 Pediatrics, Church of South India Kalyani Multispeciality Hospital, Chennai, IND

**Keywords:** adjuvant chemotherapy, primary renal angiosarcoma, radical nephrectomy, renal cell carcinoma

## Abstract

Primary renal angiosarcoma is an exceedingly rare malignancy of the kidney. It usually presents in the elderly with metastatic disease and dismal prognosis. Treatment protocols are not standardized for this very rare renal malignancy. We report the case of a young man who was incidentally diagnosed with primary renal angiosarcoma. Preoperative imaging was suggestive of renal cell carcinoma; however, postoperative histopathological examination confirmed it to be an angiosarcoma. The patient was treated with surgical excision followed by adjuvant chemotherapy and is doing well at one-year follow-up.

## Introduction

Angiosarcomas are very rare and account for 2% of the soft tissue sarcomas [1]. They arise most commonly from organs such as the skin, liver, bone, and soft tissues. Kidneys usually become involved when there is widespread systemic metastasis. The prognosis, therefore, is universally poor in such cases. Primary angiosarcoma of the kidney is exceedingly rare with only forty cases being reported in the literature. They account for around 1% of primary angiosarcomas and cannot be distinguished radiologically from renal cell carcinoma [2]. We report a case of primary renal angiosarcoma in a young male which closely mimicked renal cell carcinoma.

## Case presentation

A 42-year-old male presented to our outpatient department with an incidentally detected renal mass on ultrasonography of the abdomen done for a routine health check-up. The patient had no history of loin pain or hematuria. He never had any complaint of bone pain, loss of weight, or hemoptysis. There was no family history of malignancies. He was neither a smoker nor an alcoholic. There was no history of exposure to thorostat or vinyl chloride and he was a farmer by occupation.

**Figure 1:**
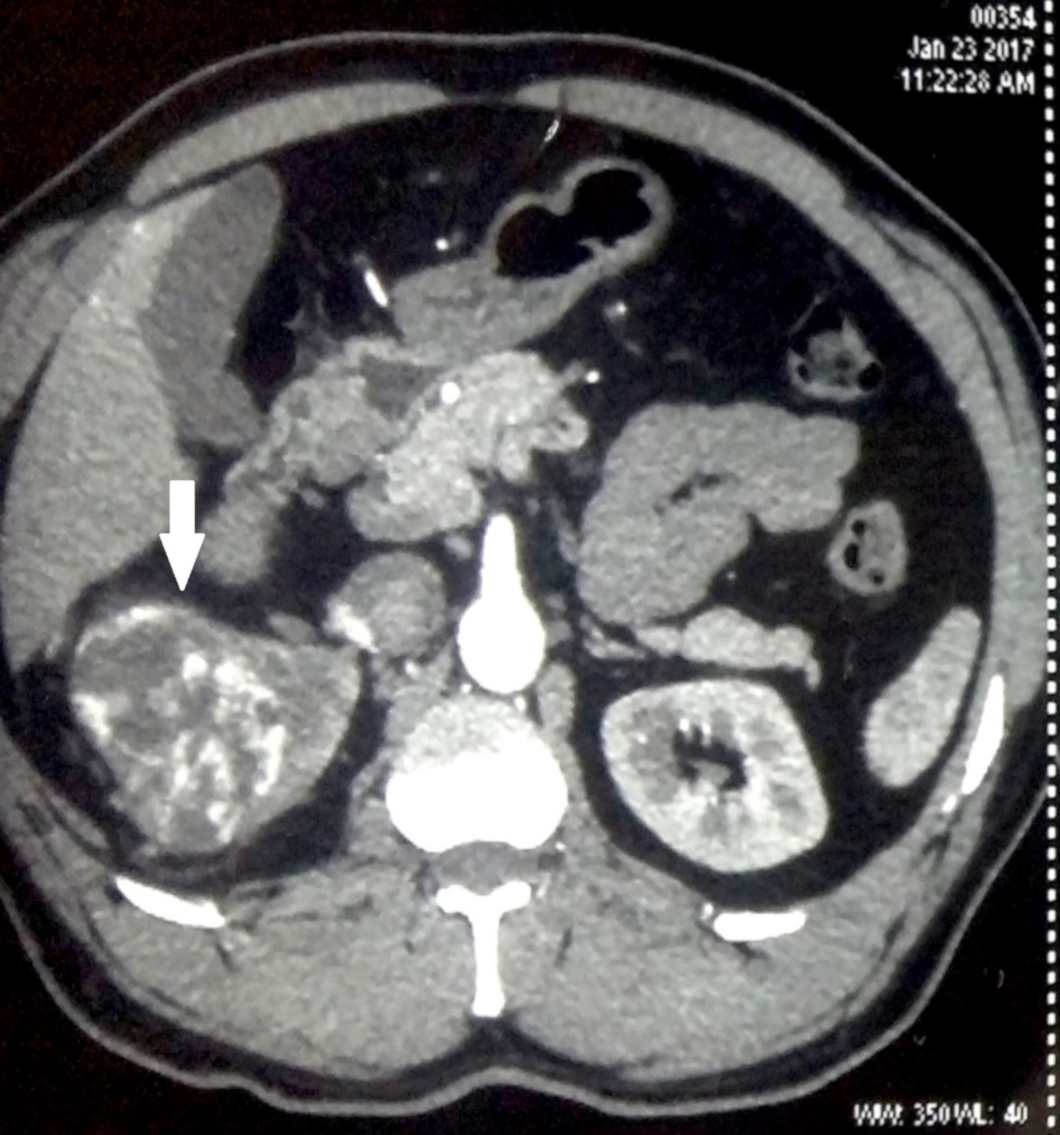
Contrast-enhanced computed tomography (CT) of the abdomen showing an enhancing heterogeneous tumor occupying the right kidney (white arrow)

**Figure 2:**
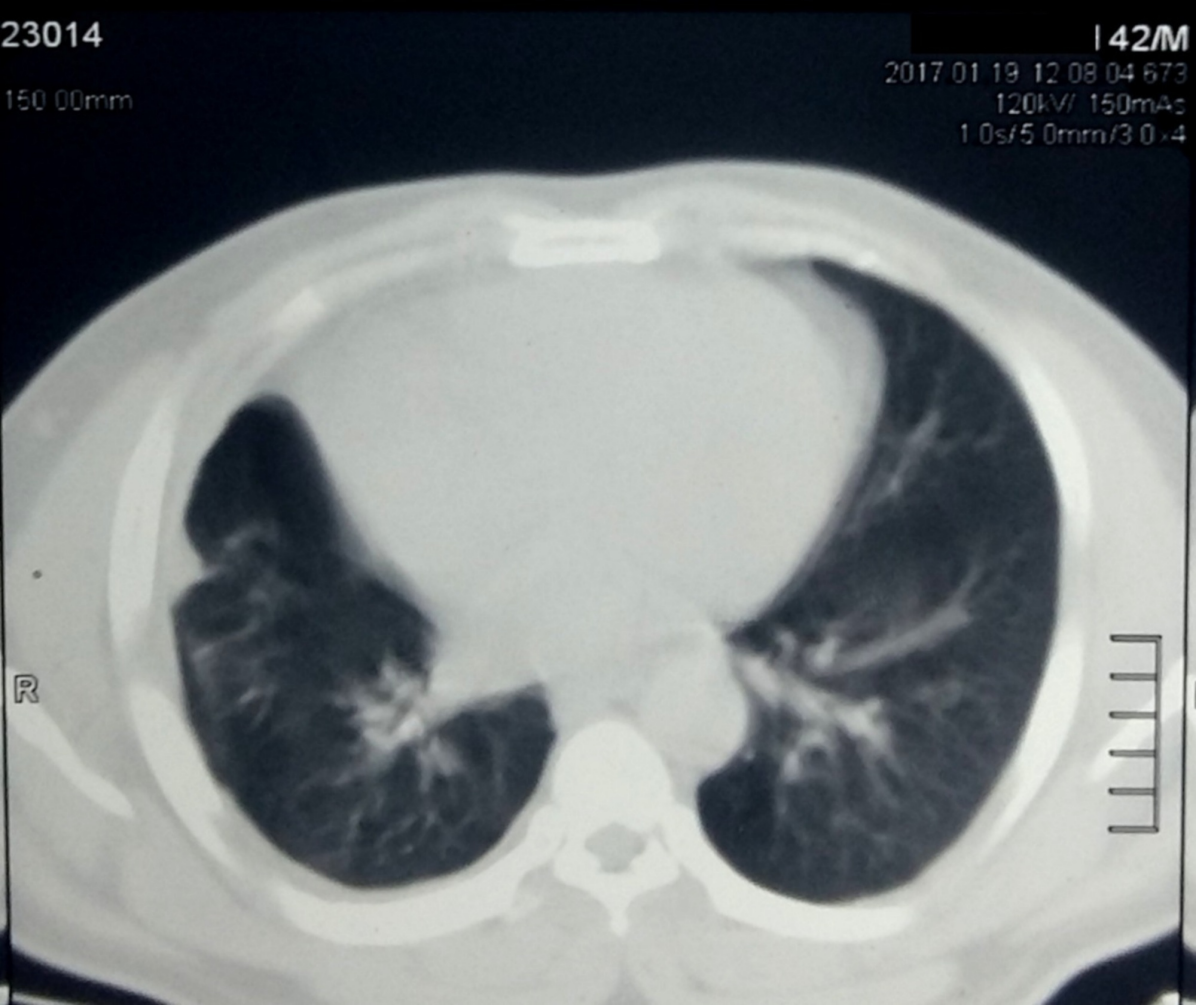
Computed tomography (CT) of the chest showing an absence of pleural or lung metastases

**Figure 3:**
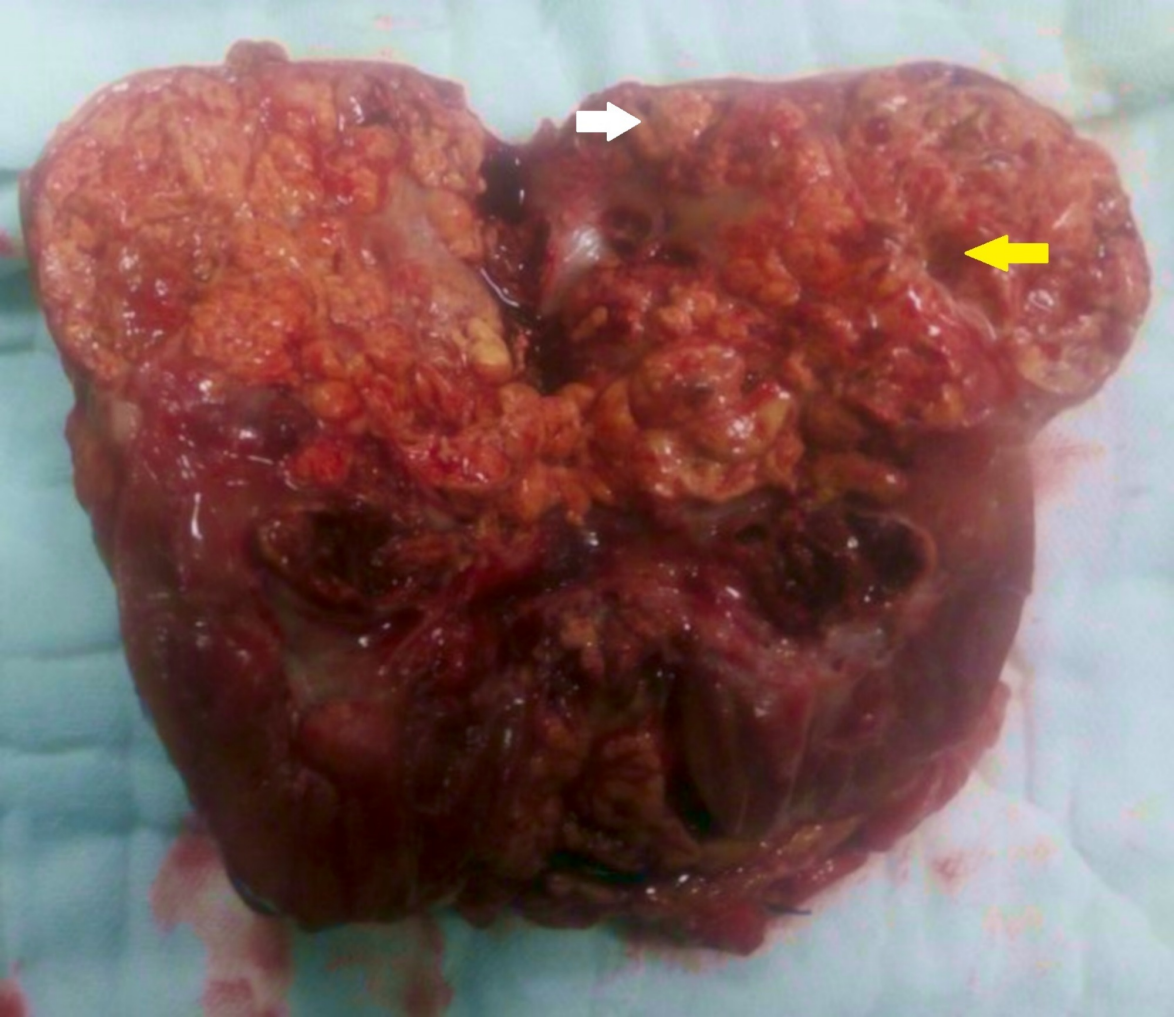
Clinical photograph of the radical nephrectomy specimen opened along the lateral renal border showing tumor involving the upper pole and interpolar region. The tumor is composed of focal fleshy (white arrow) and necrotic areas (yellow arrow) with vascular spaces

**Figure 4:**
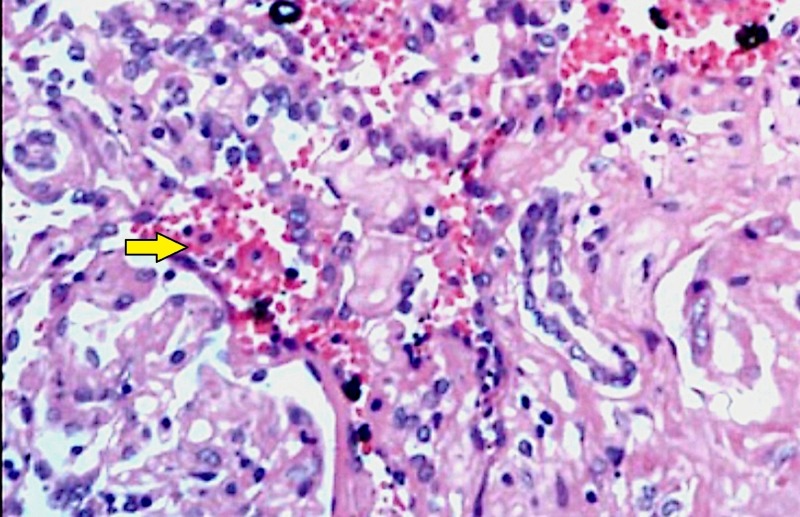
Histopathological image demonstrating the tumor with hemorrhagic areas (yellow arrow) and vascular spaces lined by cells with pleomorphic and mitotically active nuclei (hematoxylin and eosin stain, 200x magnification)

On evaluation of his hemogram, renal parameters and liver function tests were within normal limits. Contrast-enhanced computed tomography (CT) was done which revealed a 10 cm x 8 cm heterogeneous enhancing mass involving the upper pole and interpolar region of right kidney (Figure 1). The inferior vena cava and renal vein were free of thrombus and there was no regional lymphadenopathy. There was no evidence of distant metastases in the liver or bones. CT of the chest was also normal (Figure 2). The patient underwent right-sided open radical nephrectomy. On the cut section, the tumor was seen to be composed of focal fleshy and necrotic areas intermingled with vascular spaces (Figure 3). Histopathological examination revealed areas of extensive necrosis and anastomosing vascular spaces lined by pleomorphic cells (Figure 4). The tumor had a high mitotic index of 25 to 30 per ten high power fields and a ki-67 index of 40%. Immunohistochemistry was done which showed strong positivity for cluster of differentiation (CD) 34; whereas, it was negative for epithelial membrane antigen (EMA), CD 10, and human melanoma black (HMB) 45 consistent with angiosarcoma. The surgical margins were free of tumor and the pathological staging was pT2bN0M0.

In view of the histological diagnosis of angiosarcoma, the patient was treated with adjuvant chemotherapy. He received three cycles of paclitaxel-based chemotherapy. The patient is doing well on one year of follow-up. Several reports of primary renal angiosarcoma indicate a dismal outcome and predilection for the old age group. However, the present case is unique in that it occurred in a young male with a relatively better outcome which was made possible by the timely diagnosis and management.

## Discussion

Angiosarcomas are rare comprising about 2%-3% of adult soft tissue sarcomas [3]. They can occur anywhere in the body but the most common locations are skin, soft tissues, liver, and bones. Kidneys are involved as part of the widespread metastatic disease. Primary angiosarcoma of the kidney is very rarely reported and is associated with poor prognosis. Most occur in men in either the fifth or sixth decade of life [3]. The present case is peculiar in that it has presented in the early stage in a young male. Askari et al. reported a case of primary renal angiosarcoma in a renal transplant recipient presenting with systemic metastases and poor survival [4]. Around forty reports of primary renal angiosarcoma are found in literature all presenting beyond the age of 50 years and most are metastatic at presentation with poor survival. Our case represents the second youngest patient in literature; however, there was no genetic predisposition to malignancy in his family. Survival was better compared to other case reports of primary renal angiosarcoma [5-6]. Zhang et al. conducted a review of 28 cases of primary renal angiosarcoma and concluded that risk factors like thorium dioxide (thorostat), arsenic, vinyl chloride, and radiation which are predisposing factors for liver angiosarcomas can also induce primary renal angiosarcomas [3]. However, our patient never had exposure to all these chemical agents.

The predominant symptoms were flank pain, hematuria, and nonspecific symptoms in most cases. Very rarely the tumor may be incidentally detected as in our case. Akkad et al. described a case of localized primary angiosarcoma of the kidney successfully removed by surgery. The patient was not given adjuvant chemotherapy and was doing well on follow up for 30 months [7].

Preoperative diagnosis of angiosarcomas is difficult since they mimic renal cell carcinoma on imaging. They appear as enhancing heterogeneous renal tumors on contrast-enhanced CT scans. Tumor thrombosis extending into the renal vein or inferior vena cava is very rare in angiosarcoma while it is common in renal cell carcinoma. However, the only foolproof method to differentiate angiosarcoma from renal cell carcinoma is by histological examination and immunohistochemistry. Histologically the tumor is composed of anastomosing vascular spaces lined by malignant cells containing nuclei with pleomorphism and mitotic activity. Immunohistochemical staining is useful in further characterizing the tumor. It stains positive for CD 34, CD 31, and factor VIII-related antigen. Most tumor cells stain negative for CD 10, HMB 45, and EMA. Thus a panel of markers is needed to conclusively diagnose this rare renal tumor [8].

Treatment protocols are not well defined for renal angiosarcoma and hence it has to be individualized based on patient factors. Surgery forms the mainstay of treatment in most reports. Adjuvant chemotherapy is given in case of large tumors and for palliation of metastatic disease. Taxane-based chemotherapy regimens are offered to such patients though the durability of response is less [9]. The role of postoperative radiotherapy in renal angiosarcoma is controversial. Anti-angiogenesis factors like the vascular endothelial growth factor (VEGF) receptor blockers have been used successfully in the management of angiosarcoma recently and their use can be extended to renal angiosarcoma in the future [10]. The prognosis of primary renal angiosarcoma depends on the tumor size and stage at presentation. The five-year survival is 32% for tumors less than 5 cm in size compared to only 13% for those larger than 5 cm. Metastatic disease at presentation is an indicator of poor prognosis [3].

## Conclusions

This case has been presented for its rarity. Primary renal angiosarcoma can present in younger individuals with relatively better prognosis. It cannot be differentiated from renal cell carcinoma radiologically. Hence the clinician must rely on histopathological examination to confirm the diagnosis. Treatment protocols are unclear for this rare renal tumor; however, surgery and chemotherapy form the mainstay of treatment in most of the reported literature.
